# An Assessment of Behavioral Risk Factors in Oncology Patients

**DOI:** 10.3390/nu16152527

**Published:** 2024-08-02

**Authors:** Magdalena Mititelu, Monica Licu, Sorinel Marius Neacșu, Mariana Floricica Călin, Silvia Raluca Matei, Alexandru Scafa-Udriște, Tiberius Iustinian Stanciu, Ștefan Sebastian Busnatu, Gabriel Olteanu, Nicoleta Măru, Steluța Constanța Boroghină, Sergiu Lupu, Anca Coliță, Mihaela Isabela Mănescu, Carmen Elena Lupu

**Affiliations:** 1 Department of Clinical Laboratory and Food Safety, Faculty of Pharmacy, “Carol Davila” University of Medicine and Pharmacy, 020956 Bucharest, Romania; magdalena.mititelu@umfcd.ro (M.M.); gabriel.olteanu@mst.umfcd.ro (G.O.); mihaela-isabela.dragosin@mst.umfcd.ro (M.I.M.); 2 Department of Ethics and Academic Integrity, Faculty of Medicine, “Carol Davila” University of Medicine and Pharmacy, 050474 Bucharest, Romania; monica.licu@umfcd.ro; 3 Department of Pharmaceutical Technology and Bio-pharmacy, Faculty of Pharmacy, Carol Davila University of Medicine and Pharmacy, 020945 Bucharest, Romania; sorinel-marius.neacsu@drd.umfcd.ro; 4 Faculty Psychology & Educational Sciences, Ovidius University of Constanta, 900527 Constanta, Romania; mariana.calin@365.univ-ovidius.ro (M.F.C.); raluca.matei@365.univ-ovidius.ro (S.R.M.); 5 Department of Cardio-Thoracic Pathology, Faculty of Medicine, “Carol Davila” University of Medicine and Pharmacy, 050474 Bucharest, Romania; stefan.busnatu@umfcd.ro; 6 Press Office, Ovidius University of Constanța, 900527 Constanta, Romania; 7 Department of Anatomy, Faculty of Dental Medicine, “Carol Davila” University of Medicine and Pharmacy, 020945 Bucharest, Romania; nicoleta.maru@umfcd.ro; 8 Department of Complementary Sciences, History of Medicine and Medical Culture, Faculty of Medicine, “Carol Davila” University of Medicine and Pharmacy, 050474 Bucharest, Romania; steluta.boroghina@umfcd.ro; 9 Department of Navigation and Naval Transport, Faculty of Navigation and Naval Management, “Mircea cel Batran” Naval Academy, 900218 Constanta, Romania; sergiu.lupu@anmb.ro; 10 Department of Pediatrics, Faculty of Medicine, “Carol Davila” University of Medicine and Pharmacy, 050474 Bucharest, Romania; anca.colita@umfcd.ro; 11 Department of Mathematics and Informatics, Faculty of Pharmacy, “Ovidius” University of Constanta, 900001 Constanta, Romania; clupu@univ-ovidius.ro

**Keywords:** neoplastic pathology, nutritional balancing, healthy lifestyle, psychological support

## Abstract

An evaluation of the behavioral risk factors that contribute to the incidence and evolution of cancer in oncology patients was conducted through a cross-sectional study using a questionnaire completed by 206 patients (101 men and 105 women) diagnosed with various types of cancer. These patients were selected from different oncology centers in Romania, located in Bucharest and Constanta. Among the respondents, 91 are of normal weight, 12 are underweight, 62 are overweight, and 41 are obese, with overweight individuals predominating (*p* = 0.799). Regarding the presence of behavioral risk factors that can aggravate oncological pathology, it is found that 10 respondents consume alcohol daily, 36 consume it weekly with varying frequencies (*p* = 0.012), 26 respondents smoke excessively daily, and 12 respondents smoke 1–2 cigarettes daily (*p* = 0.438). Additionally, 40 respondents rarely engage in physical activity, and 71 respondents do not engage in physical activity at all as they do not typically participate in sports (*p* = 0.041). Thus, respondents with colon cancer tend to consume sweets, pastries and even fast food or fried foods more often, while the daily consumption of vegetables and fruits is insufficient, according to the recommendations of nutrition guidelines (a minimum of four portions per day). The analysis found that smoking and excessive alcohol consumption were associated with an increased incidence of lung and liver cancer. The lack of regular physical activity was identified as a risk factor for breast and colon cancer. An unhealthy diet, characterized by a low consumption of fruits and vegetables and high intake of processed foods, was correlated with a higher incidence of colorectal cancer. Additionally, non-adherence to medical advice was associated with poorer clinical outcomes and faster disease progression. The majority of respondents who declared that they did not feel an improvement in their state of health in the last period were among those who stated that they did not fully comply with the oncologist’s recommendations. Identifying and modifying behavioral risk factors can play a crucial role in cancer prevention and in improving the prognosis and quality of life of cancer patients.

## 1. Introduction

Neoplastic pathology ranks second worldwide in terms of mortality (9.3 million deaths), being surpassed only by cardiovascular diseases (17.9 million deaths) [1]. However, recent research is bringing to light results that could turn the tables for good. Due to early diagnosis through targeted interventions, cardiovascular mortality could be reduced. Unfortunately, mortality from neoplastic causes is continuously increasing and it is predicted that it could surpass cardiovascular deaths in the near future [2]. Neoplastic pathology and its treatments can adversely affect the cardiovascular system through multiple mechanisms, including direct toxicity to the heart, inflammatory and metabolic changes, and increased risk of thrombosis. The careful monitoring of cardiovascular function and preventive interventions are essential for the management of oncology patients.

According to reports available on the World Health Organization (WHO) website, an estimated 18.1 million cases of cancer were reported in 2020. Of these, 9.3 million were at-tributed to men and 8.8 million to women. In the case of men, lung cancer has the highest share (15.4% of cases), and in the case of women, breast cancer leads the way (25.8% of cases) [3].

If, in the past, it was considered that oncological pathology is the inevitable result of aging, and the genetic component plays a primary role, it seems that currently the results demonstrate the opposite [4,5]. Most cancers are generated or triggered by environmental factors and behavioral factors, including an unbalanced lifestyle that predisposes individuals to the appearance of many problems that endanger the health and proper functioning of the body [6,7,8,9,10,11,12]. Of course, when the risk factors are combined with an existing genetic predisposition to neoplasia, the risk of cancer increases. This proves once again that genes load the gun, but lifestyle pulls the trigger.

Globally, it is estimated that approximately 3–4 million cancers could be prevented each year by adopting a healthy lifestyle that is not only limited to rational and informed food choices but also includes regular physical activity, as well as the management of daily stress [13].

Thus, it is vital to know the risk factors that favorably influence the gradual progression towards oncological pathology and to combat them or reduce their detrimental effects on the body. The main factor associated with oncological pathology is smoking [14,15]. Smoking can be responsible for the appearance of many types of cancer, affecting any organ of the human body [15,16]. The risk of cancer in the head and neck region doubles when smoking is combined with alcohol consumption [17].

In second place as a modifiable risk factor for cancer is obesity, influencing both the choice of therapeutic strategy and especially mortality from neoplastic causes [18]. The specialized literature is rich in clinical studies and provides results that associate over-weight, obesity and ultimately body mass index (BMI) with the occurrence of cancers. Among many other types of cancer, obesity has been associated especially with cancer of the esophagus, breast, stomach, liver and gall bladder, pancreas, uterus, ovaries and with colon and rectal cancers [19,20,21,22,23,24,25,26,27,28,29,30,31]. In addition to the increased risk of developing cancer, obesity has also been strongly associated with an increased risk of recurrence and mortality in cancer survivors [32,33,34]. Therefore, lifestyle interventions must be adopted early, beginning from childhood so that eating habits are properly formed in adulthood and properly maintained throughout life. This is even more important since it has been shown that changes in BMI, in the sense of its decrease until approaching normal weight, are as-sociated with a decrease in mortality due to any type of cancer [35].

In fact, obesity is the result of sedentary lifestyle. Sustained physical activity per-formed regularly has been shown to be a fundamental component in the prevention of neoplastic diseases [36,37]. The WHO recommendations include at least 150–300 min of moderate-intensity physical activity and 75–150 min of vigorous-intensity physical activity per week [38]. Therefore, physical inactivity predisposes the body to a considerably higher risk of developing multiple comorbidities, including cancer.

Food choices can significantly influence the health status of each individual, and excesses in certain food categories can lead the body into a risk zone to develop multiple comorbidities. Just as there are healthy, natural foods that protect the body from cancer cells, there are also harmful, processed and ultra-processed foods that induce biochemical and physiological changes that are often irreversible or make the efforts to rebalance overwhelming.

An excess of red meat (pork, beef and sheep), an excess of foods rich in salt or pre-served in salt and also the regular consumption of processed and ultra-processed meat (sausages) are dietary behaviors associated with an increased risk of developing cancers. Thus, the Agency for Research on Cancer (IARC) classified red meat as a possible trigger of carcinogenesis, while processed meat was classified as a confirmed carcinogen [39].

Pollution significantly influences the risk of oncological diseases through continuous exposure to various chemicals and harmful particles. Often, exposure to multiple types of pollutants can have synergistic effects, further increasing the risk of cancer. For example, smoking combined with exposure to air pollution can dramatically increase the risk of lung cancer. Pollutants in food [40,41] also represent a significant risk to human health and may contribute to an increased risk of oncological conditions. These pollutants can include pesticides, heavy metals, food additives, industrial contaminants and decomposi-tion products of cooking processes [42,43,44,45]. Pollutants can induce oxidative stress, chronic inflammation and genetic changes (mutations), all of which are mechanisms by which oncological processes can be triggered. In addition, some chemicals can interfere with the normal functioning of the endocrine system, which can contribute to the development of cancer. Reducing exposure to pollutants and adopting strict environmental policies are essential to prevent cancer and protect public health.

In the context of cancer patients, the main behavioral risk factors include smoking, alcohol consumption, unhealthy diet, sedentary lifestyle, overweight and obesity, excessive exposure to ultraviolet radiation without the use of adequate sun protection, and non-compliance with medical recommendations and treatment regimens that can lead to faster disease progression and poorer clinical outcomes. In addition to behavioral factors, several intra-individual factors can significantly affect the risk of cancer. These include genetic predispositions, hormonal imbalances, immune system function, and metabolic conditions.

Despite advances in medical treatments and technology, the role of behavioral risk factors in cancer incidence, progression, and patient outcomes is often underexplored. Behavioral risk factors such as smoking, alcohol consumption, physical inactivity, and poor dietary habits are well documented contributors to cancer development. However, there is a paucity of comprehensive research examining the prevalence and impact of these factors specifically within oncology patient populations.

Addressing this evidence gap is crucial because understanding the behavioral risk profiles of oncology patients can inform tailored interventions that may improve treatment outcomes, enhance quality of life, and potentially reduce cancer recurrence rates. By assessing the behavioral risk factors in oncology patients, this research aims to fill a critical gap in the existing literature, providing insights that could lead to more holistic and effective cancer care strategies.

The study aims to identify and evaluate behavioral risk factors that contribute to the incidence and progression of cancer in oncology patients.

## 2. Materials and Methods

### 2.1. Study Design

The research was conducted on a sample of 412 patients diagnosed with different types of cancer, selected from different oncological centers in Romania (clinics and hospitals specializing in medical oncology), located in Bucharest and Constanta. Data were collected through a questionnaire between 25 February and 15 May 2024, focusing on behaviors such as smoking, alcohol consumption, diet, physical activity and adherence to medical advice. Data were analyzed using descriptive and inferential statistical methods to determine correlations between risk behaviors and disease progression. A cross-sectional study was conducted using a validated 56-item questionnaire, designed to ensure the accuracy and reliability of the data collected. This questionnaire has been rigorously tested for reliability and validity, meaning it consistently produces accurate measurements and effectively captures the intended constructs. Details of the validation process, including the methods used to assess reliability and validity, are provided in the Supplementary Materials section. The collection of the following data was pursued: age, sex, marital status, anthropometric data (height, weight), level of education, status occupational, food habits, and lifestyle. Based on the anthropometric data, the body mass index (BMI) was calculated [46].

The inclusion criterion of the respondents was represented by age (from 18 years and above) and the presence of oncological conditions. This study specifically included only patients who are currently undergoing treatment for their cancer: colon cancer, gastric cancer, liver cancer, pancreatic cancer, prostate cancer, lung cancer, kidney cancer, breast cancer, thyroid cancer and others (most of them being different types of leukemia). Patients who had completed their therapy and were off treatment were excluded. By focusing on patients actively undergoing treatment, this study aims to provide a relevant and timely assessment of behavioral risk factors, contributing to the development of targeted interventions that may improve patient outcomes during their cancer treatment journey.

The dissemination of the questionnaire was achieved through specialized staff from the oncology centers, the Google Forms platform was used to centralize the answers, for the security settings related to the protection of sensitive data (blocking the collection of respondents’ identification data) and also for the settings that ensured accuracy and consistency answers (blocking the collection of multiple answers). Participants in this study signed informed consent forms before their inclusion in the survey. This process ensured that all participants were fully informed about the study’s objectives and methods and voluntarily agreed to take part. By obtaining informed consent, the study adheres to ethical standards and respects participants’ autonomy and rights.

The study was conducted in accordance with the Declaration of Helsinki and approved by the Ethics Commission of the Carol Davila University of Medicine and Pharmacy from Bucharest, no. 4201/16 February 2024.

### 2.2. Statistical Analysis

Categorical variables were described using frequencies (n) and percentages (%). The chi-square test was used to assess the independence of various categorical variables such as smoking habits, dietary patterns, and adherence to medical treatments across different cancer types. Following the chi-square tests, multiple comparison analysis (MCA) was performed to further explore the differences between groups [47]. This analysis helped in identifying specific patterns and significant differences among the subgroups within the variables tested.

All statistical analyses were conducted using XLSTAT (version 2020, Addinsoft, New York, NY, USA), which facilitated robust data handling and advanced statistical computations. Statistical significance was set at *p*-values less than 0.05.

## 3. Results

### 3.1. Socio-Demographic Characteristics and Lifestyle

A total of 520 oncology patients were contacted to participate in this survey study. These patients were selected based on their current status of undergoing cancer treatment at various oncology clinics and hospitals. The selection process was designed to ensure a representative sample of the oncology patient population, considering factors such as age, gender, and type of cancer. Out of the 520 patients contacted, 108 did not participate in the study. Reasons for non-participation included a lack of interest, time constraints, and health-related issues that prevented participation. Consequently, 412 patients completed the survey, resulting in a response rate of approximately 79%. Of the 412 respondents participating in the study, 49.1% are male and 50.9% female, 38.5% of the respondents are between 36 and 71 years old (*p* = 0.1900), 80.6% come from urban areas (*p* = 0.4000), 55.8% have higher education (*p* = 0.604), 33.5% are retired, 38.3% are employed, 7.8% are students (*p* = 0.6040), 44.2% are normal weight, 49.9% are overweight or obese (*p* = 0.799), 62.6% are married (*p* = 0.0260), 76.2% are non-smokers (*p* = 0.438), 77.7% consume alcohol very rarely or not at all (*p* = 0.012), 48.6% are sedentary or do not usually conduct physical activity at all (*p* = 0.041), 77.7% have a normal, omnivorous diet (*p* = 0.525), and 38.4% had antecedents of oncological pathology in the family. The respondents have various oncological diseases, and among the most common are the following: 18.4% lung cancer, 13.1% breast cancer, 11.2% gastric cancer, 11.2% colon cancer, 9.7% pancreatic cancer, 9.2% kidney cancer, and 8.7% prostate cancer. Only 15% of the respondents engage in daily physical activity (*p* = 0.0409), the majority only engage in 30 min of it (11.6%), 19.4% engage in sports rarely, and 38.6% do not engage in sports. The main reason for physical inactivity is represented by the tendency towards sedentarism (34.5% of the respondents do not engage in sports due to the fact that they are not used to carrying out sports activities), and 14.1% do not engage sports because their state of health does not allow them. Women are more active than men in the group of study participants. Regarding the rest period, 46.1% of respondents sleep 7–9 h per night, 15.1% have frequent insomnia, and 35.4% sleep less than 7 h per night. Although over 76% of the respondents do not smoke and do not consume alcohol, considering that we have associated smoking and alcohol consumption with oncological pathology, the processed data indicate that 12.6% of the respondents smoke daily and excessively, 5.8% smoke 1–2 cigarettes daily, and 5.4% smoke occasionally. Regarding alcohol consumption, 4.9% of respondents consume alcohol daily (*p* = 0.012), and 17.4% consume alcohol weekly (once, twice or more than twice) (Table 1).

**Figure 1:**
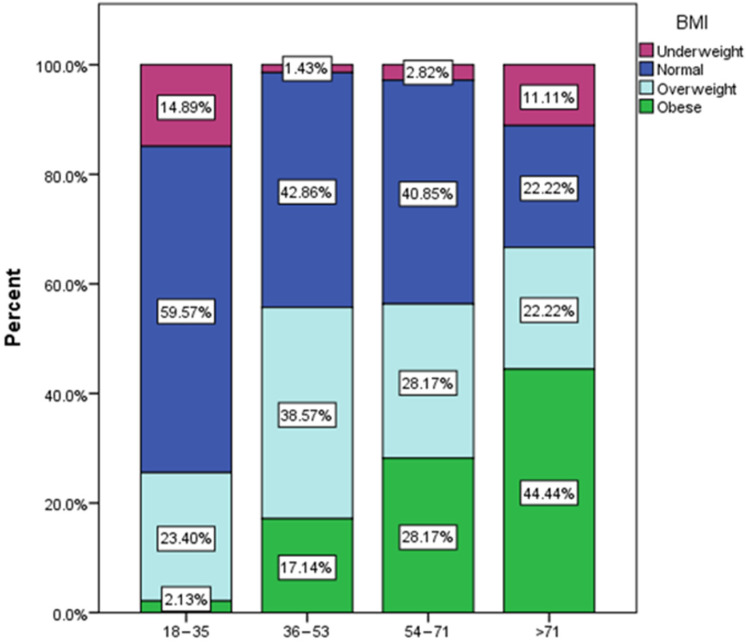
Distribution of BMI by age groups.

Figure 1 shows the distribution of BMI groups according to age group. Most of the normal weight (59.57%) and underweight respondents (14.89%) are among the young respondents (18–35 years old), and most of the obese (44.44%) are among respondents over 71 years old.

**Figure 2:**
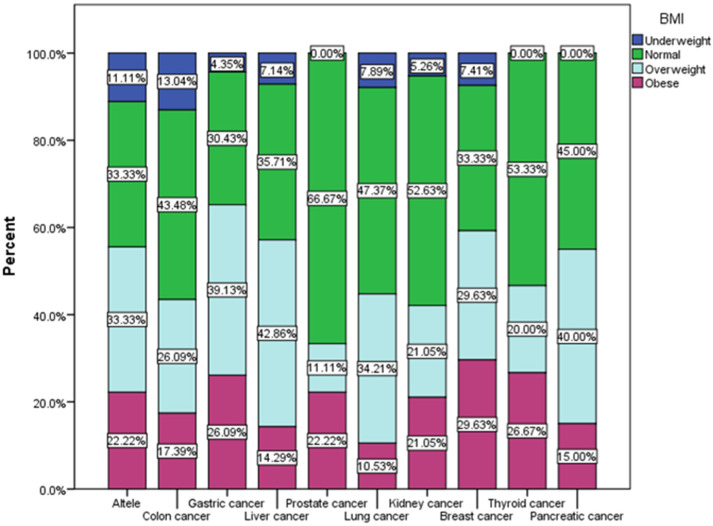
Type of cancer by BMI groups (χ^2^ = 21.33, *p* = 0.074).

According to the data in Figure 2, we observe that in each category of oncological pathology, normal weight respondents predominate, constituting the majority of those with prostate cancer (66.67%, χ^2^ = 21.33, *p* = 0.074) and kidney cancer, but in each category of pathology in oncology, we also have overweight respondents, comprising the majority of those with liver cancer (42.86%), pancreatic cancer (40%) and gastric cancer (39.13%), and also obese, comprising the majority among those with breast cancer (29.63%), thyroid cancer (26.67%) and gastric cancer (26.09%).

### 3.2. Eating Habits and Lifestyle

**Figure 3:**
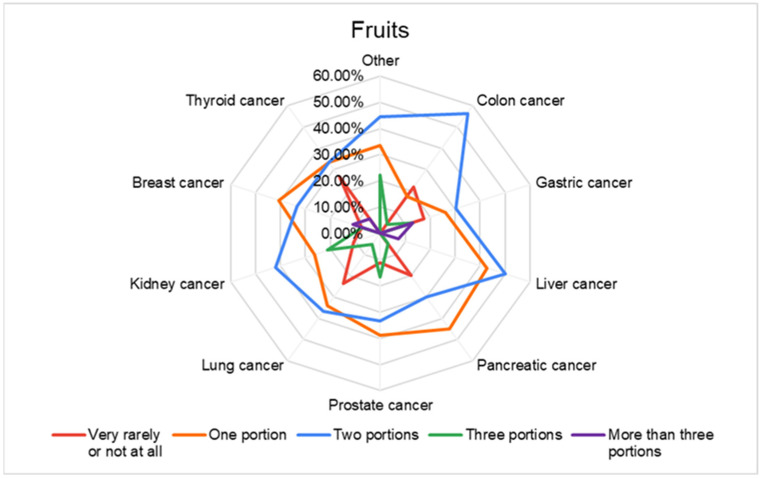
Fruit consumption in different types of cancer.

From Figure 3, we observe that, in general, the respondents consume one or two servings of fruit per day, and a higher consumption is noted in respondents with colon cancer, pancreatic cancer and breast cancer.

**Figure 4:**
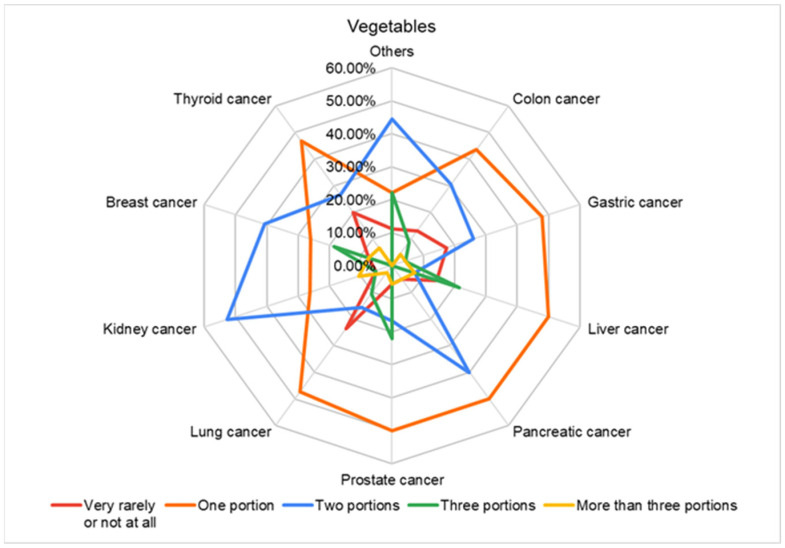
Vegetable consumption in different types of cancer.

Figure 4 highlights a relatively low consumption also in terms of vegetable products, so the statistical processing of the data indicated a consumption tendency among the respondents of a single portion of vegetables per day and a higher consumption (two portions of vegetables per day) among respondents with colon cancer and liver and kidney cancer.

We quantified the frequency of consumption sweets, pastries, sweetened and carbonated drinks and alcohol beverages, and the following quantification of consumption frequency was used: 5—very rare or no consumption; 4—consumption 2–3 times a month; 3—consumption once a week; 2—consumption 2–3 times a week; and 1—daily consumption of one portion (for sweets and pastries, a 100 g portion, and for beverage, 330 mL/portion). We noted the following: alc—alcoholic beverages, s-dr—sweetened drinks, and sw/pas—sweets/pastries.

**Figure 5:**
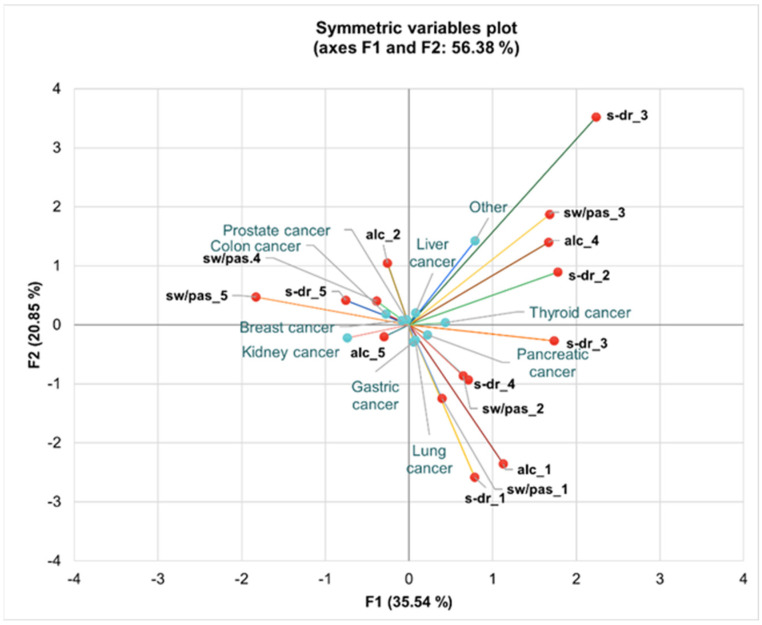
Multiple correspondence analysis (MCA) for associations between healthy dietary habits and types of cancer across axes F1 and F2, representing 56.38% of total variance. Legend: alc—alcoholic beverages, s-dr—sweetened drinks, sw/pas—sweets/pastries; code 1–5 associated with the consumption frequency.

Patients with lung and gastric cancer are frequently associated with the daily consumption of alcoholic beverages and sweetened drinks, as indicated by the proximity of alc_1 and s-dr_1 in the negative region of F1 (Figure 5).

Patients with breast and kidney cancer show a different consumption pattern. These patients are linked to very rare or no consumption of sweets/pastries and alcoholic beverages, as seen by the placement of sw/pas_5 and alc_5 towards the positive extremities of F1 and F2 (Figure 5). This implies a minimal intake of these potentially harmful dietary elements, which could be part of a health-conscious approach following diagnosis.

Furthermore, individuals with prostate and colon cancer appear to consume sweets/pastries and alcoholic beverages 2–3 times a month, positioned slightly towards the central and positive sections of the axes. This indicates a moderate but infrequent consumption pattern among these patients.

Liver and thyroid cancer patients, located near the markers for the weekly consumption of alcoholic beverages (a portion), reflect a habitual but controlled dietary intake of alcohol.

The quantification of the consumption of vegetables, fruits, pasta, rice or other grains, fish or sea food and dairy products was conducted as follows: 5—the consumption of more than three portions of 100 g per day; 4—the consumption of three portions per day; 3—the consumption of two portions per day; 2—the consumption of one portion per day; and 1—consumption very rarely or no consumption. We noted the following: veg—vegetable products; fru—fruits; fish/sf—fish or sea food; dai—dairy products; and pas/rice—pasta or rice.

**Figure 6:**
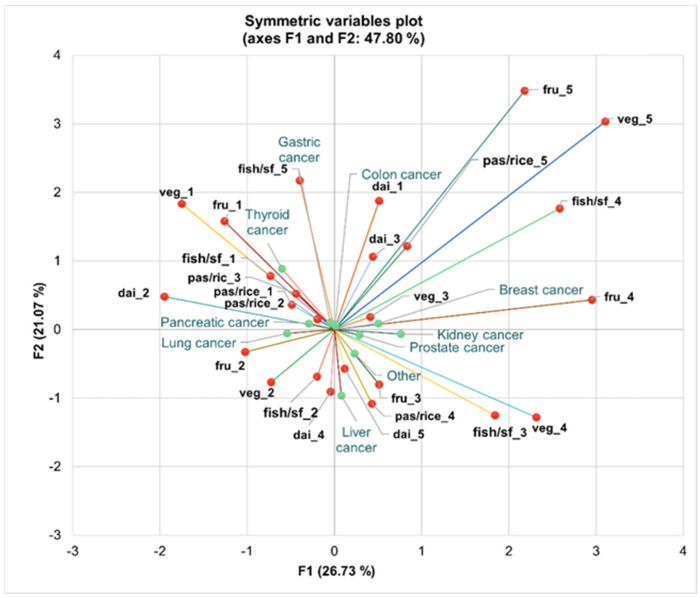
Multiple correspondence analysis (MCA) for associations between healthy dietary habits and types of cancer across axes F1 and F2, representing 47.80% of total variance. Legend: veg—vegetable products; fru—fruits; fish/sf—fish or sea food; dai—dairy products; pas/rice—pasta or rice; code 1–5 associated with the consumption frequency.

Patients with lung and pancreatic cancer are characterized by very rare or no consumption of fruits and vegetables, as shown by the proximity of fru_1 and veg_1 in the negative region of F1 (Figure 6).

Individuals diagnosed with breast and colon cancer demonstrate a high consumption pattern, linked to eating more than three portions per day of vegetables and pasta and rice, as demonstrated by the position of veg_5 and pas/rice_5 towards the positive extremities of both axes.

Additionally, patients with thyroid and gastric cancer show consumption patterns associated with three portions per day of fish or sea food and dairy products, particularly seen with fish/sf_4 and dai_4.

Liver cancer patients are depicted near the markers for daily single portion consumption, particularly with fish/sf_2, suggesting a moderate, routine consumption of fish/seafood.

According to the data presented in Table 2, 31.1% of the respondents declared that, in the last period, they presented an improvement in their state of health, 25.2% declared that they had a deterioration in their state of health, and 43.7% declared that they had experienced no change in health. Most of the respondents consume home-cooked meals 86.7% (*p* = 0.0342). Although healthy cooking methods (boiling, steaming, and cooking in the oven) predominate as options, 22.2% of the respondents declared that they mainly consume foods cooked by frying. Also, related to the consumption of liquids, plain water or natural juices predominate (60%), but 14.4% mainly consume carbonated or sweetened non-alcoholic beverages, and 11.1% consume large amounts of coffee (*p* = 0.0784). A disordered schedule of meals was also noted; very few respondents declared that they had a fixed schedule for meals, and the majority of those who did declared that they presented a worsening of their health in the last period, having a disordered schedule related to the distribution of meals during of the day.

Based on the processed responses, the following negative aspects related to the quality of the respondents’ diet were noted: 44.7% of the respondents primarily use refined vegetable oil as dietary fat, 22.8% mainly use butter, and only 28.2% consume virgin or extra virgin vegetable oil; 41.7% of respondents consume a single portion of approximately 100 g of vegetables per day, 29.1% two portions, only 10.7% consume three portions per day, 5.3% more than three portions per day, and 13.1% consume vegetables very rarely or not at all; 33.5% consume only one portion of fruit per day of approximately 100 g, 38.3% consume two portions of fruit per day, 8.7% consume three portions a day, 3.9% more than three portions, and 15.5% consume fruit very rarely or not at all; 15% consume sweets or pastries daily and 31.1% consume sweets or pastries more than twice a week; 10.2% consume carbonated or sweetened drinks daily; 36.4% consume fish or seafood very rarely or not at all, 45.1% consume them only once a week; and 27,1% consume approximately 1 L of water per day and 6.8% less than 1 L.

It is interesting that 45.1% of the respondents stated that they do not know if their diet has affected their health, 6.8% stated that they are aware of the fact that they consume unhealthy food, 9.2% are aware of the fact that they consume an excessive amount of food, and 7.8% declared that they consume insufficient amounts of food. Only 0.5% of the respondents declared that they consume food according to rations established by a nutrition specialist, 34% declared that they consume food chaotically, without adhering to a structured routine or a support system that promotes digestion, 26.2% eat in a hurry, while 19.9% tend to multitask during meals.

Only 8.3% of the respondents with oncological conditions participating in the study declared that they adopted a Mediterranean diet, 2.9% adopted a vegetarian diet, 1% a vegan diet, and interestingly, 59.7% of the respondents believe that they needed the advice of a nutritionist to improve the quality of their diet.

Alongside their oncological treatment, a significant portion of respondents also used nutritional supplements (27.2%) and natural treatments (23.3%). Regarding access to the oncologist’s services, 23.8% of the respondents declared that they can get to the doctor on the same day if needed, 45.1% can get there within a few days, 6.3% can get there in a week, and 24.8% can see a doctor in more than a week. Regarding the way in which they comply with the oncologist’s instructions, 18.4% admitted that they only partially comply, the rest declaring that they fully comply. Most of the respondents spend a large part of their free time especially in front of the television or on social networks; moreover, 16.5% declared that they spend more than 8 h in front of the screens (computer, television, or phone), 11.7% declared that they spend about 6–7 h a day, 26.5% spend about 4–5 h a day, while only 12.7% spend less than an hour.

**Figure 7:**
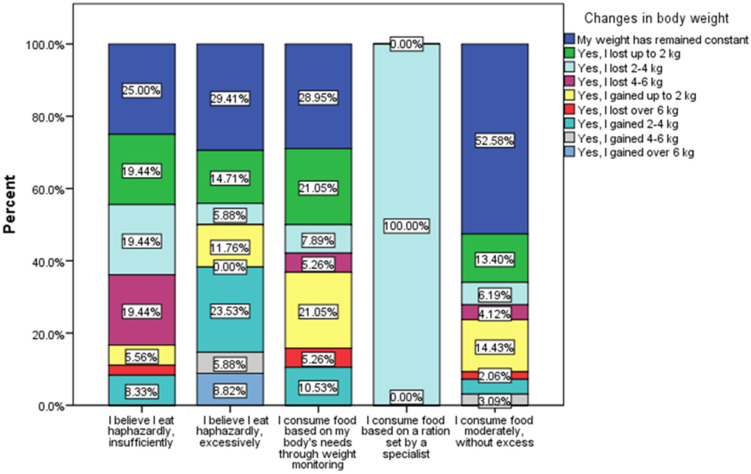
Quantity of food of daily consumed and changes in body weight (χ^2^ = 61.28, *p* < 0.0001).

The respondents who turned to specialists in order to have a balanced food consumption adapted to the needs of the body do not have large fluctuations related to body weight (Figure 7); they all presented a weight loss of 2–4 kg, and all the others presented various changes in terms of regarding body weight or, in different proportions, a maintenance of body weight, the highest percentage of respondents who kept their body weight unchanged (52.58%) being in the group of those who declared that they used to consume food in moderation (χ^2^ = 61.28, *p* < 0.0001).

**Figure 8:**
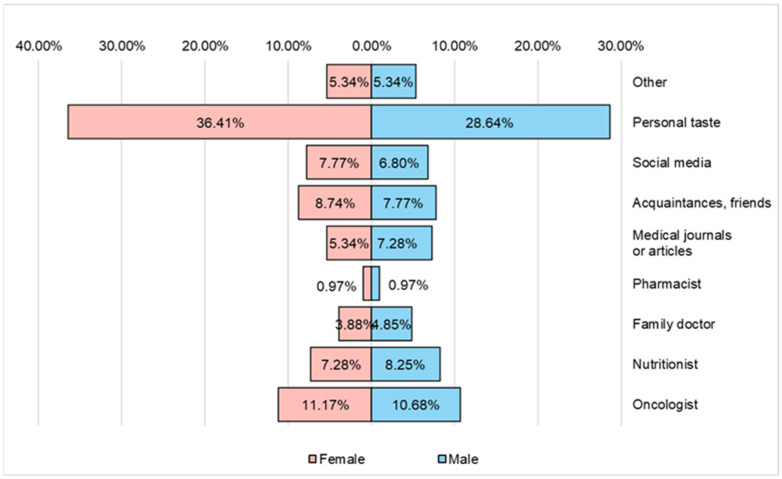
Ways of choosing food products from the diet and/or food supplements used by the respondents.

According to the data presented in Figure 8, the respondents participating in the study declared that food products and food supplements were chosen according to personal tastes (36.41% of women and 28.64% of men), a small percentage of respondents turn to the advice of a nutritionist (7.28% of women and 8.25% of men) or to the advice of an oncologist (11.17% of women and 10.68% of men).

**Figure 9:**
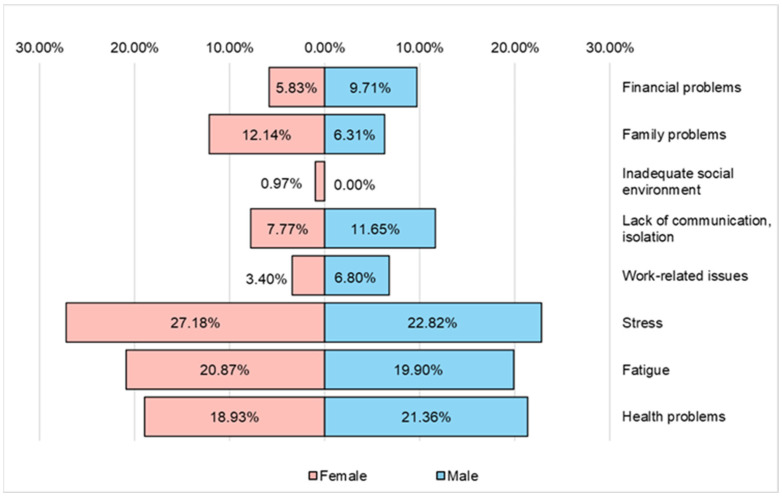
The main factors affecting the mental state of the respondents.

The main factors that affect the mental state of respondents with oncological conditions participating in the study are (Figure 9) stress, fatigue and health problems. Female respondents (27.18%) are more stressed than male respondents (22.82%).

**Figure 10:**
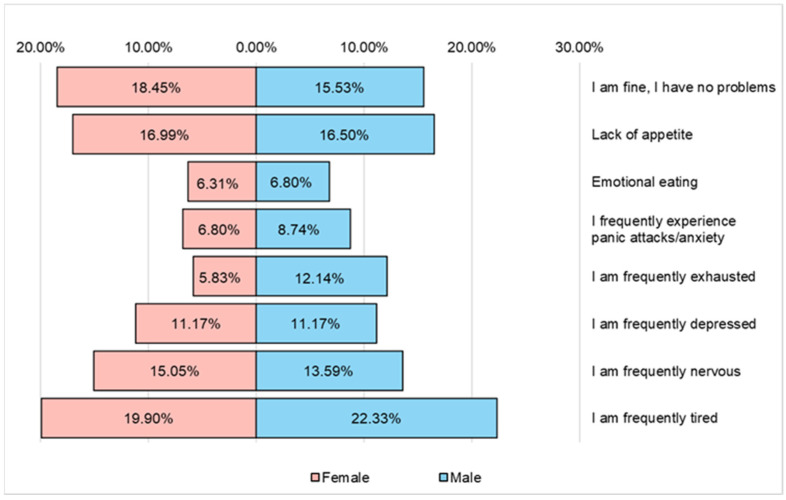
The main types of problems encountered by respondents that affect their quality of life.

According to the answers received from the respondents, the most frequent problems that affect their quality of life are fatigue, nervousness, depression and a lack of appetite (Figure 10).

**Figure 11:**
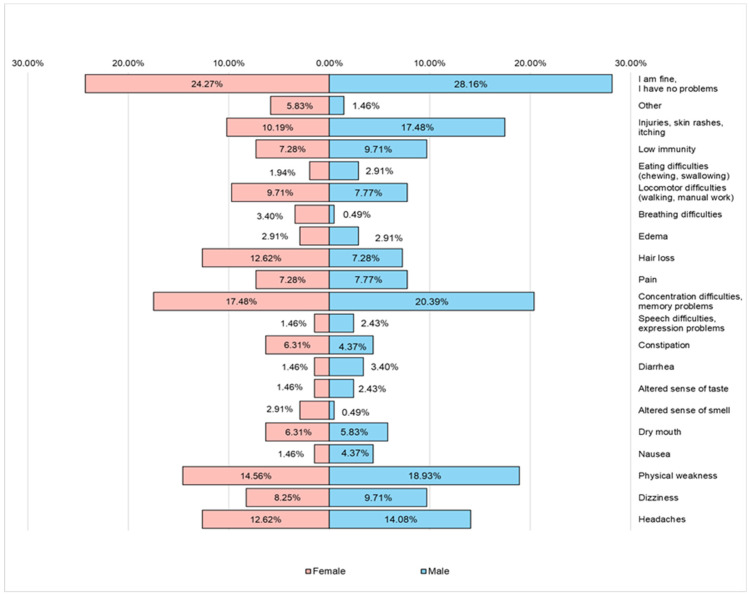
The type of clinical manifestations most often faced by the respondents in the last period.

The main clinical manifestations most often faced by the respondents in the last period are represented by (Figure 11) injuries, skin eruptions, itching, difficulties in concentration, memory problems, states of physical weakness, and headaches (especially male respondents are more affected by these disturbing clinical manifestations).

**Figure 12:**
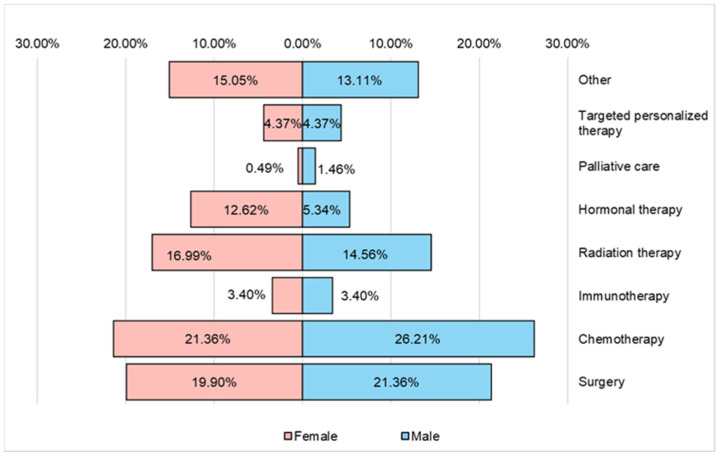
The type of therapy followed by the respondents.

Figure 12 shows the main types of therapy followed by respondents with oncological conditions participating in the study. Chemotherapy, surgery and radiotherapy predominate. From the group of respondents, a very small percentage benefited from targeted personalized therapy.

**Figure 13:**
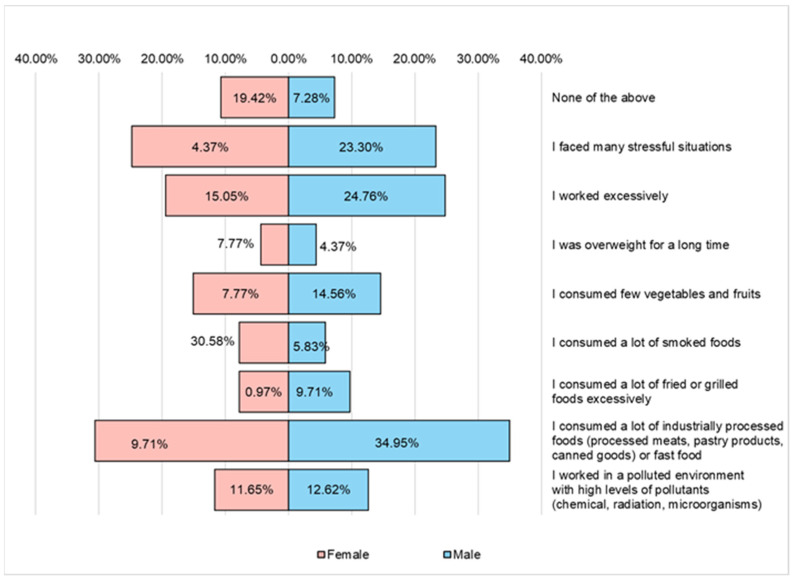
The main risk factors for health that the respondents faced in the past.

In the case of male respondents, the main health risk factors they faced in the past are represented by (Figure 13) the increased consumption of industrially processed foods (sausages, pastries, and canned goods) or fast food products (34.95%), excessive work (24.76%) and stressful situations (23.30%). In the case of female respondents, excessive work (15.5%) and work in environments with high pollution (11.65%) are noteworthy.

## 4. Discussion

The study included a total number of 412 respondents participating with oncological diseases. Overweight and obese respondents predominate, most of them having breast and colon cancer. Excess weight can be correlated with reduced physical activity, many of the respondents with excess weight falling into the category of those who show an increased tendency towards sedentarism. Many of those who do not rest enough are not used to engaging in sports, the tendency towards sedentarism being one of the causes. Regarding physical activity, oncology patients are encouraged to adopt a physical exercise program under the guidance of a specialist (physiotherapist). Physical activity exerts beneficial effects both in primary prevention and also in secondary and tertiary prevention. Physical activity can reduce adverse effects of treatment and has beneficial effects on exercise tolerance (reduced fatigue), mobility, stability, balance and mental health [48]. Patients can be included in cardiac rehabilitation programs, knowing that antineoplastic therapy can cause cardiotoxicity [49,50,51]. Therefore, it is necessary for the patient to be trained in terms of physical activity, guided, supervised and encouraged throughout the process in order to gain confidence in the specialist and especially in their own strength. Another worrisome aspect is the consumption of alcohol and smoking in the group of respondents. A greater consumption tendency regarding alcohol is noted among male respondents. The consumption of alcohol and tobacco is higher among the respondents with lung and liver cancer, and it is possible that the respondents had these habits before the onset of the oncological pathology and did not manage to give up these unhealthy habits.

The OECD [52] report on health in Romania, which discusses the distribution of dis-eases and the main health indicators, highlights that oncological pathology represents the second leading cause of mortality (after cardiovascular diseases), accounting for approximately 16.7% of all deaths in Romania. The types of cancer with the highest incidence rates are prostate, lung, colorectal and breast cancer. Life expectancy in Romania was 75.3 years in 2022, being one of the lowest in the EU. The decline in life expectancy was influenced by the COVID-19 pandemic, which reduced life expectancy by about 0.3 years. Major risk factors include high rates of smoking and heavy drinking compared to other EU states. Approximately 35% of adults in Romania reported excessive alcohol consumption, almost double the EU average. Five-year survival rates for treatable cancers are well below the EU average, including prostate cancer (77% compared to 87% in the EU) and breast cancer (75% compared to 82% in the EU) [52]. We could also highlight from the results of our study that there are patients with oncological diseases who do not give up unhealthy habits such as tobacco and alcohol consumption even after the diagnosis of the pathology, a fact that can endanger the health of patients and even their lives.

Although, the clinical cases encountered in oncology departments in hospitals around the world are mostly cachectic, anorexic patients (most of the time as a side effect of the therapeutic scheme and also of decreased appetite and an abnormal assimilation of nutrients and reconfiguration of metabolism) [53,54], the obesity phenotype may still be present in those patients who do not stabilize in terms of lifestyle determinants. Obesity is identified as a risk factor in 4–8% of cancers and significantly increases the risk of death from neoplastic causes, increases the risk of cancer recurrence, and influences choices regarding therapeutic approaches (obesity status increases chemotherapy toxicity and predisposes individuals to surgical complications) [55,56].

The health status of a cancer patient is in continuous degradation. However, there are many patients who do not comply with the recommendations in terms of lifestyle changes and the adoption of practices that improve symptoms and progressive cellular changes throughout the entire spectrum of biological organization (cells–tissues–organs–organ systems). The presence of obesity, even after cancer diagnosis, in the context of hyper-caloric and low-nutritional-density food intake, combined with inefficient nutrient absorption due to oncological therapy, increases fragility and may predispose individuals to major cardiovascular events [57]. It is well known that obesity is an independent risk factor for cardiovascular disease in the absence of a cancer diagnosis [58]. Lifestyle changes are the first approach to minimize the risk of cardiotoxicity. This approach primarily involves quitting smoking, limiting alcohol consumption to a maximum of 100 g per week, adopting a rational and balanced diet that meets the body’s needs, and importantly engaging in regular physical activity under the guidance of a physiotherapist and as recommended and prescribed by a specialist doctor [59]. In fact, a healthy and balanced lifestyle reduces the risk of cancer, cardiovascular disease, and the transition from diagnosed neoplastic pathology to an associated cardiac pathology [60].

Similar to imbalances in food intake, certain patients do not give up habits they had before being diagnosed with cancer, namely the consumption of tobacco (in any form, cigarettes, e-cigarettes, and snuff) and alcohol (and not infrequently, even in considerable quantities) [61]. These behavioral factors impose a significant risk in the progression of neoplasia, as well as in interactions with treatment regimens for oncology patients and in the recurrence among survivors [61]. Smoking status prior to diagnosis increases the risk of mortality, especially in the case of smokers consuming more than 60 packs per year, being 5.4 (95% CI= 0.7–40.1) [62]. Regarding alcohol consumption prior to diagnosis, the situation is similar for those patients who consumed more than five drinks per day, the risk being 4.9 (95% CI = 1.5–16.3) [62]. Obviously, for those patients who did not address these factors following the diagnosis of cancer (so, post diagnosis), the chances of survival were significantly reduced [62].

In the fight against oncological diseases, various organizations worldwide have been involved, such as the World Health Organization, the European Society for Oncology Medicine, the American Society of Clinical Oncology, the European Coalition of Cancer Patients (ECPS) and many other patient associations organized at the global or local level. These associations are fighting a constant battle both in promoting the most useful and targeted treatment methods and, as much as possible, a personalized approach to every case of oncological patients. Additionally, they work on propagating guidelines and recommendations to accompany the patients’ treatments and to prevent the appearance of cancer in the global population. Thus, the World Health Organization even issued a code called the European Cancer Prevention Code, which has several rules in its structure that can help to avoid the occurrence of cancer. The recommendations of this guide are addressed both to the general population and to the authorities in each country. The involvement of the authorities in this endeavor is vital for the success of the project. The World Health Organization recognizes that a united fight carried out by the individual is insufficient if it is devoid of the involvement of the authorities [63].

The guide issued by the World Health Organization places major emphasis on the fight against smoking addiction and suggests that blocking the sale of tobacco could significantly change the evolution of cancer cases. Additionally, it advocates for promoting access to a healthy lifestyle by encouraging sports activities and healthy foods, thereby creating an environment conducive to eliminating the risk of cancer. Regarding nutrition, it is recommended to increase the intake of vegetables and fruits along with fiber from whole grains and to avoid processed foods such as meat with excess salt used in its processing or the so-called “empty calories” that come from foods rich in sugar and fats [63].

An alternative for balanced nutrition for a patient with oncological conditions could be the Mediterranean diet [64]. A diet rich in plant fibers from vegetables and fruits, legumes, kernels, seeds and also whole grains is extremely useful in maintaining nutritional balance by providing omega 3 fatty acids. In addition to these types of food, a moderate intake of fish products, milk and eggs is recommended. Due to its structure, the Mediterranean diet provides a much-needed balance during cancer treatment and can be considered for continued use afterward to prevent relapses [65,66].

It is vital that nutritionists be active members of a multidisciplinary cancer care team. The nutritional care process for cancer patients begins with nutritional screening. The results of our study highlighted a reduced collaboration of respondents with oncological conditions with nutrition specialists. It can be observed that there are problems related to body weight, eating healthy foods, and giving up unhealthy habits (smoking, alcohol consumption, and physical inactivity), which can significantly affect the quality of life and the recovery of the respondents.

An insufficient intake of macronutrients and micronutrients, essential for the good functioning of the body, and also of fibers, through a reduced consumption of fruits, vegetables and whole grains, predisposes the cells of the body to the harmful effects of free radicals and pathogens from the external environment. The inadequate hydration of the body can affect the state of health and especially the detoxification capacity of the body. A dietary deficiency of iron, zinc, iodine, calcium, and vitamins A, B6, B9, B12, C, and D has been associated with an increased risk of carcinogenesis [67,68,69,70,71,72]. Also, chronic alcohol consumption produces harmful effects in the body (toxic metabolites, reactive oxygen species, and pro-inflammatory status) correlated with increased cancer risk [73,74].

Environmental factors, namely atmospheric pollution, play an important role in the process of carcinogenesis. The main source of pollution is radon, a natural gas present in soil and water, which is responsible for 3%–14% of lung cancers in various countries [75,76]. In addition to this, there are many other substances found in the occupational environment, which can increase the risk of cancer, such as arsenic, asbestos, exhaust gas emission from engines and some forms of silicon and chromium [77]. In the group of respondents participating in the study, exposure to pollutants from professional environments was also found as a potential risk factor in the development of oncological pathology.

An oncological patient requires an integrative, multidisciplinary and personalized approach. The nutrition of an oncological patient is very important because an altered nutritional status can compromise oncological therapy by reducing the patient’s tolerance to the treatment regimen. In their diet, emphasis should be placed on foods rich in vitamins (especially folate) and antioxidants, i.e., fruits and vegetables and whole grains, as well as quality protein sources as it is known that cancer patients lose muscle mass and enter a state of cachexia. Therefore, it is imperative that a multidisciplinary team includes a registered dietitian or a nutritionist to ensure the necessary macronutrients and micronutrients, as well as to provide recommendations on food preparation and nutritional education information to patients. With the help of nutritional therapy, a patient with cancer can tolerate chemotherapy and radiotherapy much better, reduce the adverse effects of the procedures and ensure a higher quality of life. Adequate nutrition during treatment for cancer patients is like a secret weapon, helping to support the body’s strength and resistance in the fight against cancer.

Psychological support is essential for cancer patients and significantly contributes to improving their quality of life, emotional state and clinical outcomes. A cancer diagnosis can cause anxiety, depression and other psychological disorders. Psychological support helps to reduce these conditions through counseling and cognitive behavioral therapy. Psychological counseling can motivate patients to follow recommended treatment plans and adhere to their therapeutic schedule, which is particularly important for treatment effectiveness. Psychological intervention can help manage physical and emotional symptoms such as pain, fatigue and insomnia. Patients receive help in managing relationships with family and friends, which may be affected by the disease, and in maintaining a strong social support system. Psycho-oncology therapists can facilitate open and effective communication between patient and family, helping to express needs and emotions. Psycho-oncologists can help patients make informed decisions about treatment and care based on a clear understanding of the available options and personal preferences. A positive emotional state and stress reduction can have beneficial effects on the immune system, contributing to a better response to treatment. Also, the effective management of stress and emotions can reduce the risk of psychosomatic complications such as hypertension and cardiovascular disease.

### Limitations of the Study

Within the study, there are a series of limitations primarily due to the number of respondents. For a more relevant analysis of the influence of behavioral risk factors in oncological disease, it is necessary to carry out similar studies on the largest possible groups. Another limitation arises from the uneven distribution of different types of oncological diseases among the respondents, highlighting the need for studies that encompass a diverse range of oncological conditions. Additionally, there is a possibility of subjectivity in the responses, particularly regarding sensitive topics like alcohol consumption and smoking, which respondents may underreport based on advice from specialist doctors to avoid exacerbating their health conditions.

## 5. Conclusions

This study emphasizes the critical role of behavioral changes in cancer prevention and management. Interventions aimed at reducing smoking, alcohol consumption, improving diet, increasing physical activity, and enhancing medical advice adherence can significantly reduce the risk and improve the prognosis of cancer patients. Smoking and excessive alcohol consumption are strongly associated with increased incidences of lung and liver cancer. Targeted interventions to reduce these behaviors can lower the risk and potentially improve outcomes for affected patients. An unhealthy diet, characterized by a low intake of fruits and vegetables and a high consumption of processed foods, is linked to a higher incidence of colorectal cancer. Encouraging dietary changes to align with nutritional guidelines can reduce risk factors and support overall health. A lack of regular physical activity is a risk factor for breast and colon cancer. Programs promoting regular exercise can play a crucial role in prevention and should be integrated into patient care plans. Non-adherence to oncologists’ recommendations is associated with poorer clinical outcomes and faster disease progression. Enhancing treatment adherence through patient education and support can lead to better health outcomes and an improved quality of life.

The implementation of health education and psychological support programs, along with public policies aimed at reducing behavioral risk factors, is essential to enhance the quality of life and clinical outcomes for cancer patients. Psychological support plays a crucial role in the emotional and psychological well-being of cancer patients, with positive impacts extending to clinical outcomes and the overall quality of life.

In conclusion, identifying and addressing behavioral risk factors is crucial in cancer prevention and patient management. By adopting a comprehensive approach that includes lifestyle modifications, psychological support, and adherence to medical guidelines, we can significantly improve the prognosis and quality of life for cancer patients. A holistic approach to cancer care that integrates behavioral risk modification, psychological support, and patient education is essential for enhancing the quality of life and clinical outcomes for cancer patients. Collaborative efforts among healthcare providers, policymakers, and community organizations are vital to implementing these strategies effectively.

## Figures and Tables

**Table 1:** Socio-demographic anthropometric characteristics and lifestyle of respondents (n = 412).

Variables	Total Population n (%)	Male n (%)	Female n (%)
	412 (100)	202 (49.1)	210 (50.9)
**Age (years)**		***p* = 0.190**	
18–35	94 (22.8)	48 (23.8)	46 (21.9)
36–53	140 (34.0)	62 (30.7)	78 (37.1)
54–71	142 (34.5)	66 (32.7)	76 (36.2)
>71	36 (8.7)	26 (12.9)	10 (4.8)
**Residence areas**		***p* = 0.400**	
Urban areas	332 (80.6)	158 (78.2)	174 (82.9)
Rural areas	80 (19.4)	44 (21.8)	36 (17.1)
**Level of education**		***p* = 0.604**	
General/primary studies	40 (9.7)	22 (10.9)	18 (8.6)
Secondary education (baccalaureate degree)	90 (21.9)	46 (22.8)	44 (21.0)
Post-secondary studies	52 (12.6)	22 (10.9)	30 (14.3)
Higher education (bachelor’s degree)	146 (35.4)	64 (31.7)	82 (39.0)
Postgraduate studies (master’s degree, residency, doctorate, other specializations)	84 (20.4)	48 (23.7)	36 (17.1)
**Employment status**		***p* = 0.604**	
Medical leave	46 (11.2)	26 (12.9)	20 (9.5)
Socially assisted	6 (1.4)	2 (1.0)	4 (1.9)
Householder	32 (7.8)	10 (5.0)	22 (10.5)
Retired	138 (33.5)	68 (33.6)	70 (33.3)
Student/school student	32 (7.8)	20 (9.9)	12 (5.7)
I go to work every day	146 (35.4)	70 (34.6)	76 (36.2)
I work in a mixed regime (telework and commuting)	12 (2.9)	6 (3.0)	6 (2.9)
**Body mass index (BMI)**		***p* = 0.799**	
Normal weight (18.5–24.9)	182 (44.2)	96 (47.5)	86 (41.0)
Overweight category (25–29.9)	124 (30.1)	58 (28.7)	66 (31.4)
Underweight category (<18.5)	24 (5.8)	10 (5.0)	14 (6.6)
Obese (≥30)	82 (19.9)	38 (18.8)	44 (21.0)
**Marital status**		***p* = 0.026**	
Single	98 (23.8)	64 (31.7)	34 (16.2)
Divorced/separated	56 (13.6)	28 (13.9)	28 (13.3)
Married	258 (62.6)	110 (54.4)	148 (70.5)
**Smoke habits**		***p* = 0.438**	
Yes, occasionally	22 (5.4)	16 (7.9)	6 (2.9)
Yes, 1–2 cigarettes daily	24 (5.8)	12 (5.9)	12 (5.7)
Yes, excessively daily	52 (12.6)	26 (12.9)	26 (12.4)
No	314 (76.2)	148 (73.3)	166 (79.0)
**Alcohol consuming**		***p* = 0.012**	
Twice a week	18 (4.3)	10 (5.0)	8 (3.8)
Very rarely or not at all	320 (77.7)	154 (76.2)	166 (79.0)
More than two times a week	14 (3.4)	10 (5.0)	4 (1.9)
Once a week	40 (9.7)	10 (5.0)	30 (14.3)
One serving daily	20 (4.9)	18 (8.9)	2 (1.0)
**Sleep habits**		***p* = 0.421**	
I frequently have insomnia	62 (15.1)	24 (11.9)	38 (18.1)
Less than 7 h per night	146 (35.4)	74 (36.6)	72 (34.3)
More than 9 h per night	14 (3.4)	10 (5.0)	4 (1.9)
7–8 h per night	190 (46.1)	94 (46.5)	96 (45.7)
**Sports habits**		***p* = 0.041**	
Yes, 30 min daily	48 (11.6)	18 (8.9)	30 (14.3)
Yes, at least one hour daily	18 (4.4)	10 (4.8)	8 (3.8)
Yes, 2–3 times a week	58 (14.1)	30 (14.9)	28 (13.3)
Yes, once a week	8 (1.9)	6 (3.0)	2 (1.0)
Yes, very rarely	80 (19.4)	28 (13.9)	52 (24.8)
No, due to health conditions	54 (13.1)	30 (14.9)	24 (11.4)
No, because I am immobilized	4 (1.0)	2 (1.0)	2 (1.0)
No, because I do not usually exercise	142 (34.5)	78 (38.6)	64 (30.4)
**Type of diet**		***p* = 0.525**	
Normal omnivorous diet	320 (77.7)	162 (80.2)	158 (75.2)
Vegetarian diet/variations	12 (2.9)	6 (3.0)	6 (2.9)
Vegan diet/variations	4 (1.0)	0 (0.0)	4 (1.9)
Mediterranean diet	34 (8.2)	10 (8.9)	16 (7.6)
Other	42 (10.2)	16 (7.9)	26 (12.4)
**Antecedents of oncological pathology in the family**		***p* = 0.348**	
Yes	158 (38.4)	68 (33.7)	90 (42.9)
No	172 (41.7)	88 (43.5)	84 (40.0)
I do not know	82 (19.9)	46 (22.8)	36 (17.1)
**Type of cancer**		*p* < 0.0001	
Colon	46 (11.2)	24 (11.9)	22 (10.5)
Gastric	46 (11.2)	24 (11.9)	22 (10.5)
Liver	28 (6.8)	18 (8.9)	10 (4.8)
Pancreatic	40 (9.7)	24 (11.9)	16 (7.6)
Prostate	36 (8.7)	36 (17.8)	0 (0.0)
Lung	76 (18.4)	40 (19.9)	36 (17.1)
Kidney	38 (9.2)	16 (7.9)	22 (10.5)
Breast	54 (13.1)	0 (0.0)	54 (25.7)
Thyroid	30 (7.3)	10 (4.9)	20 (9.5)
Other	18 (4.4)	10 (4.9)	8 (3.8)

**Table 2:** Dietary habits and the evolution of health in the recent period (n = 412).

Variables	Nothing Changed n (%)	It Depreciated n (%)	It Improved n (%)
	180 (43.7)	104 (25.2)	128 (31.1)
**Category of food products**		***p* = 0.0342**	
Home-cooked meals	156 (86.7)	94 (90.4)	120 (93.8)
Food cooked in restaurants	16 (8.9)	6 (5.8)	4 (3.1)
Pizza, snacks, pastry, sweets	2 (1.1)	4 (3.8)	2 (1.6)
Fast food products	4 (2.2)	0 (0.0)	0 (0.0)
Products made from processed meats and canned goods	2 (1.1)	0 (0.0)	2 (1.6)
**Type of cooked foods**		***p* = 0.0435**	
Boiled or steamed foods	66 (36.7)	44 (42.3)	46 (35.9)
Grilled foods	12 (6.7)	10 (9.6)	4 (3.1)
Raw or minimally processed foods	4 (2.2)	0 (0.0)	4 (3.1)
Fried foods	40 (22.2)	20 (19.2)	20 (15.6)
Oven-baked foods	56 (31.1)	26 (25.0)	50 (39.1)
Foods cooked over wood or charcoal	2 (1.1)	4 (3.8)	4 (3.1)
**Category of liquids**		***p* = 0.0784**	
Still water and natural juices	108 (60.0)	70 (67.3)	80 (62.5)
Carbonated or sweetened non-alcoholic beverages	26 (14.4)	8 (7.7)	8 (6.2)
Coffee	20 (11.1)	22 (21.2)	28 (21.9)
Tea	26 (14.4)	4 (3.8)	12 (9.4)
**Meal distribution**		***p* = 0.0876**	
I consume 1–2 meals per day without a fixed schedule	74 (41.1)	26 (25.0)	36 (28.1)
I consume 3 meals per day without a fixed schedule	16 (8.9)	4 (3.9)	16 (12.5)
I consume 3 meals per day and 1–2 snacks without a fixed schedule	70 (38.9)	38 (36.5)	40 (31.2)
I consume 3 meals per day on a fixed schedule	6 (3.3)	10 (9.6)	24 (18.8)
I consume 3 meals per day and 1–2 snacks on a fixed schedule	14 (7.8)	26 (25.0)	12 (9.4)

## Data Availability

The original contributions presented in the study are included in the article, and further inquiries can be directed to the corresponding author.

## Supplementary Materials

The following supporting information can be downloaded at: https://www.mdpi.com/article/10.3390/nu16152527/s1, Survey: Assessment of behavioral risk factors in oncology patients. References [78,79,80] are cited in the supplementary materials.
