# Supplementary material for: An Assessment of Behavioral Risk Factors in Oncology Patients
Source: Nutrients. 2024 Aug 2;16(15):2527. doi: 10.3390/nu16152527 (PMC11314614; doi:10.3390/nu16152527)
Supplement: Supplementary file 1 [file nutrients-16-02527-s001.zip › nutrients-3103758-supplementary.pdf]

Supplementary material

QUESTIONNAIRE: Assessment of Behavioral Risk Factors in Oncology Patients

Informed consent

Dear Sir/Madam, if you agree with your participation in completing the following questionnaire, which is part of a study initiated by the Carol Davila University of Medicine and Pharmacy in Bucharest, a study that aims to evaluate the Assessment of Behavioral Risk Factors in Oncology Patients, please accept the completion of the questionnaire after carefully reading the terms and conditions. The questionnaire is set in such a way as not to collect the identification data of the respondents, to prevent the collection of multiple answers from the same user and to protect personal data according to Regulation (EU) 2016/679 of the European Parliament and of the Council of 27 April 2016 on the protection of natural persons with regard to the processing of personal data and on the free movement of such data, and repealing Directive 95/46/EC (General Data Protection Regulation).
I agree that the answers collected through the questionnaire will be statistically processed and published in scientific articles. I agree to participate in completing the questionnaire without financial compensation, on a voluntary basis.
By completing the questionnaire, I agree with the established terms and conditions.

Personal Information
1. Please specify your age (in years):

2. Please indicate your gender:
   Male
   Female

3. Please specify your current place of residence:
   City
   Village/Town

4. Please mention your marital status:
   Single
   Divorced/Separated
   Married

5. Please indicate your level of education:
   Elementary/Primary School
   Secondary School (High School Diploma)
   Vocational/Technical School
   Higher Education (Bachelor's Degree)
   Postgraduate Education (Master's, Residency, PhD, other specializations)

6. Please specify your occupational status:
   Unemployed
   Social Assistance Recipient

Homemaker
Retired
Student/School Student
Medical Leave
Remote work
Commute to Workplace Daily
Mixed Work Arrangement (Remote Work and On-Site Work)

Anthropometric Data
7. Please state your weight (in kilograms):

8. Please state your height (in centimeters):

Dietary Habits:

9. What is the primary type of dietary fat that you consume?
Margarine
Lard, bacon fat
Butter
Refined vegetable oil
Extra virgin or virgin vegetable oil

10. How many servings of vegetables (approximately 100g each) do you consume daily?
Very rarely or not at all
One
Two
Three
More than three

11. How many servings of fruits (approximately 100g each) do you consume daily?
Very rarely or not at all
One
Two
Three
More than three

12. How often do you consume meat?
Very rarely or not at all
Once a week
Twice a week
More than twice a week
Daily

13. How often do you consume carbonated or sweetened beverages (1 serving = 330 ml, one glass)?
Daily
More than twice a week
Twice a week
Once a week
Very rarely or not at all

14. How often do you consume alcoholic beverages (1 glass of wine = 125ml, 1 glass of spirits = 50ml)?
Daily, more than one serving

Daily, one serving
More than twice a week
Twice a week
Once a week
Very rarely or not at all

15. How often do you consume fish or seafood?
Very rarely or not at all
Once a week
Twice a week
More than twice a week
Daily

16. How often do you consume sweets/pastry products?
Daily
More than twice a week
Twice a week
Once a week
Very rarely or not at all

17. How often do you consume pasta, rice, or other grains?
Very rarely or not at all
Once a week
Twice a week
More than twice a week
Daily

18. How much bread do you consume per day?
More than 12 slices
8-12 slices
5-7 slices
1-4 slices
Very rarely or not at all

19. How often do you consume dairy products?
Very rarely or not at all
Once a week
Twice a week
More than twice a week
Daily

20. How many eggs do you consume per week?
Very rarely or not at all
1 - 2 eggs
3 - 4 eggs
5 - 7 eggs
More than 7 eggs

21. What category of food products do you consume most frequently?
Fast food products
Pizza, snacks, pastry, sweets
Products made from processed meats and canned goods

Food cooked in restaurants
Home-cooked meals
Special dietary or therapeutic food purchased from specialized units

22. What type of cooked foods do you consume most frequently?
Fried foods
Foods cooked over wood or charcoal
Grilled foods
Oven-baked foods
Boiled or steamed foods
Raw or minimally processed foods

23. How much water do you drink per day?
Less than 1 liter
1 liter
2 liters
3 liters
More than 3 liters

24. What category of liquids do you most commonly consume?
Alcoholic beverages: sparkling wine, beer, etc.
Carbonated or sweetened non-alcoholic beverages
Coffee
Tea
Still water and natural juices

25. Which category of food products predominates in your daily diet? (Multiple options)
Vegetables and fruits
Fish and seafood dishes
Cereals and pasta
Dairy products
Meat and meat products
Sweets and pastry products
High-fat foods
Special foods for cancer patients

26. What type of meat do you consume most frequently?
Fish and/or seafood
Poultry
Rabbit meat
Beef
Game meat
Pork
Lamb meat
Goat meat
Other
I do not consume meat

27. The choice of food products in your diet and/or dietary supplements is based on information received from: (Multiple options)
Oncologist
Nutritionist

Family doctor
Pharmacist
Medical journals or articles
Acquaintances, friends
Social media
Personal taste
Other

Lifestyle:

28. How are your meals distributed throughout the day?
I consume 1-2 meals per day without a fixed schedule
I consume 3 meals per day without a fixed schedule
I consume 3 meals per day and 1-2 snacks without a fixed schedule
I consume 3 meals per day on a fixed schedule
I consume 3 meals per day and 1-2 snacks on a fixed schedule

29. How do you assess the quantity of food you consume daily?
I believe I eat haphazardly, excessively
I believe I eat haphazardly, insufficiently
I consume food moderately, without excess
I consume food based on my body's needs through weight monitoring
I consume food based on a ration set by a specialist

30. Do you believe your diet has affected your health?
I don't know
Yes, because I consume unhealthy foods
Yes, because I overeat
Yes, because I undereat
No

31. How do you approach mealtime?
I generally eat in a hurry
During meals, I tend to do other things as well
I eat my meal calmly and without rushing

32. Do you think you need advice from a nutritionist? (Multiple options)
Yes, to help me choose healthy foods
Yes, to help me eat moderately
Yes, because I need to lose weight urgently
Yes, because I need to gain weight urgently
No

33. What type of diet are you currently following?
Normal omnivorous diet
Vegetarian diet/variations
Vegan diet/variations
Ketogenic diet
Mediterranean diet
Other

34. In the last 3 months, have you experienced any changes in your body weight?

Yes, I lost up to 2 kg
Yes, I lost 2-4 kg
Yes, I lost 4-6 kg
Yes, I lost over 6 kg
Yes, I gained up to 2 kg
Yes, I gained 2-4 kg
Yes, I gained 4-6 kg
Yes, I gained over 6 kg
My weight has remained constant

35. Do you currently smoke?
Yes, excessively daily
Yes, 1-2 cigarettes daily
Yes, 2-3 times a week
Yes, occasionally
No

36. How many hours per night do you usually sleep?
I frequently have insomnia
Less than 7 hours per night
More than 9 hours per night
7-8 hours per night

37. Have you been engaging in physical exercise recently?
Yes, 30 minutes daily
Yes, at least one hour daily
Yes, 2-3 times a week
Yes, once a week
Yes, very rarely
No, due to health conditions
No, because I am immobilized
No, because I do not usually exercise

38. What do you consider to be the main factors affecting your mental state? (Multiple options)
Health problems
Fatigue
Stress
Work-related issues
Lack of communication, isolation
Inadequate social environment
Family problems
Financial problems
None

39. What kind of issues do you face? (Multiple options)
I am frequently tired
I am frequently nervous
I am frequently depressed
I am frequently exhausted
I frequently experience panic attacks/anxiety
Emotional eating
Lack of appetite

I am fine, I have no problems

40. What type of clinical manifestations do you most frequently experience and are most bothersome to you lately? (Multiple options)

Headaches
Dizziness
Physical weakness
Nausea
Dry mouth
Altered sense of smell
Altered sense of taste
Diarrhea
Constipation
Speech difficulties, expression problems
Concentration difficulties, memory problems
Pain
Hair loss
Edema
Breathing difficulties
Locomotor difficulties (walking, manual work)
Eating difficulties (chewing, swallowing)
Low immunity
Injuries, skin rashes, itching
Other
I am fine, I have no problems

41. Which of the clinical manifestations have been tolerable for you in the recent period among those listed? (Multiple options)

Headaches
Dizziness
Physical weakness
Nausea
Dry mouth
Altered sense of smell
Altered sense of taste
Diarrhea
Constipation
Speech difficulties, expression problems
Concentration difficulties, memory problems
Pain
Hair loss
Edema
Breathing difficulties
Locomotor difficulties (walking, manual work)
Eating difficulties (chewing, swallowing)
Low immunity
Injuries, skin rashes, itching
I am fine, I have no problems

42. Which of the clinical manifestations have not bothered you at all in the recent period among those listed? (Multiple options)

Headaches

Dizziness
Physical weakness
Nausea
Dry mouth
Altered sense of smell
Altered sense of taste
Diarrhea
Constipation
Speech difficulties, expression problems
Concentration difficulties, memory problems
Pain
Hair loss
Edema
Breathing difficulties
Locomotor difficulties (walking, manual work)
Eating difficulties (chewing, swallowing)
Low immunity
Injuries, skin rashes, itching
I am fine, I have no problems

43. How do you assess the evolution of your health in the recent period?
It has deteriorated
It has improved
There has been no change

44. What type of therapy have you undergone or are currently undergoing? (Multiple options)
Surgery
Chemotherapy
Immunotherapy
Radiation therapy
Hormonal therapy
Palliative care
Targeted personalized therapy
Other

45. What other treatments have you tried besides oncologic treatment? (Multiple options)
Nutritional supplements
Naturopathic treatments
Acupuncture
Yoga
Others
No other treatment

46. How much time do you spend on average each day in front of a computer, tablet, phone, or television?
More than 8 hours
6-7 hours
4-5 hours
2-3 hours
Less than 1 hour

47. What other types of chronic conditions do you have? (Multiple options)
Hypertension

Cardiovascular diseases
Diabetes
Obesity
Lung diseases
Kidney conditions
Autoimmune diseases
Gastric conditions
Liver conditions
Respiratory conditions
Rheumatic conditions
Bone conditions
Mental health conditions
Other
No other conditions
I don't know

48. Which of the following situations have you encountered in the past? (Multiple options)
I worked in a polluted environment with high levels of pollutants (chemical, radiation, microorganisms)
I worked with asbestos products or in an asbestos factory
I had excessive sun exposure or used procedures for maintaining a tan
I smoked moderately or excessively
I consumed alcohol moderately or excessively
I used drugs moderately or excessively
I consumed a lot of industrially processed foods (processed meats, pastry products, canned goods) or fast food
I consumed a lot of fried or grilled foods excessively
I consumed a lot of smoked foods
I consumed a lot of carbonated beverages
I consumed few vegetables and fruits
I was overweight for a long time
I worked excessively
I faced many stressful situations
I abused medications
None of the above

49. Have you had family members diagnosed with cancer?
Yes
No
I don't know

50. What do you think is currently lacking in your life to improve your lifestyle? (Multiple options)
Free time
Restful sleep, relaxation
Financial resources
Guidance from a nutrition specialist
Psychological counseling
Access to quality medical services
Resolution of stressful problems
Communication and socialization
Reducing time spent on social media or various websites
Knowledge in the field of nutrition
Exercise, physical activity

Quality food products
Someone to help me whenever I need it
Nothing

51. How are your oncologist appointments provided?

Free through the public healthcare system

For a fee through the private healthcare system

Both modalities

52. How easily can you reach your doctor from the moment you contact them?

On the same day

Within a few days

Within a week

More than a week

53. To what extent do you follow your current doctor's recommendations?

Completely

Partially

54. How often do you have a medical consultation?

Monthly

Every 6 months

Every 3 months

Annually

More than a year

55. How do you spend your leisure time? (Multiple options)

Watching movies and TV programs

On social media or various websites

Reading

DIY projects

With family or friends

Outdoor activities

Participating in cultural activities

Participating in charitable events

Participating in training courses

Other ways

55. What type of oncological pathology do you have? (one option)

Lung cancer

Liver cancer

Gastric cancer

Breast cancer

Colon cancer

Pancreatic cancer

Prostate cancer

Kidney cancer

Thyroid cancer

Other

Questionnaire and scoring key

For 56 questionnaire items, scores were assigned [1-3] to each response based on association with healthy dietary habits and healthy lifestyle. Higher points were given to responses with healthy dietary habits and healthy lifestyle, lower points were given to responses with bad dietary habits and lifestyle habits.
The information collected to assess the different indicators is shown below:
Diet: Questions were formulated referring to the frequency of consuming of the different food groups, the amount of liquid and the amount of soft drinks.
Management of Treatment Side Effects: Involve questions about how patients cope with and manage the side effects related to cancer treatments, such as nausea, hair loss, and fatigue.
Quality of Life and Mental Health: This subscale focus on psychological well-being and social support
Treatment Compliance: questions which evaluate how well patients adhere to prescribed medical treatments and lifestyle adjustments recommended by their healthcare providers.
Oncological Health Monitoring: This includes regular screenings, follow-up appointments, and self-monitoring practices that are vital for managing the illness effectively.

Personal data:

1. Please mention your age (in years): ……………………..

2. Please mention your gender:

Male	1
Female	2

3. Please mention your currently reside:

City	1
Village/Town	2

4. Please state your marital status:

Single	1
Divorced /Separated	2
Married	3

5. Please indicate your level of education:

Elementary/Primary School	1
Secondary School (High School Diploma)	2
Vocational/Technical School	3
Higher Education (Bachelor's Degree)	4
Postgraduate Education (Master's, Residency, PhD, other specializations)	5

6. *Please specify your occupational status:*

Unemployed	1
Social Assistance Recipient	2
Homemaker	3
Retired	4
Student/School Student	5
Medical Leave	6
Remote work	7
Commute to Workplace Daily	8
Mixed Work Arrangement (Remote Work and On-Site Work)	9

Anthropometric data

7. Please mention your weight (in kilograms): …………

8. Please state your height (in centimeters): ………..

BMI was assessed according to the World Health Organization criteria and defined as a person's weight in kilograms divided by their squared height in meters (kg/m^2).

Dietary Habits:

9. What is the primary type of dietary fat that you consume?

Margarine	1
Lard, bacon fat	2
Butter	3
Refined vegetable oil	4
Extra virgin or virgin vegetable oil	5

10. How many servings of vegetables (approximately 100 each) do you eat every daily?

Very rarely or not at all	1
One	2
Two	3
Three	4
More than three	5

11. How many servings of fruits (approximately 100 g each) do you eat consume daily?

Very rarely or not at all	1
One	2
Two	3
Three	4
More than three	5

12. How often do you consume meat?

Very rarely or not at all	1
Once a week	2
Twice a week	3
More than 2 times a week	5
Daily	4

13. How often do you consume carbonated or sweetened beverages (1 serving = 330 ml, one glass)?

Daily	1
More than twice a week	2
Twice a week	3
Once a week	4
Very rarely or not at all	5

14. How often do you consume alcoholic beverages (1 glass of wine=125ml, 1 glass of soft spirits =50ml)?

Daily, more than one serving	1
Daily, one serving	2
More than twice a week	3
Twice a week	4
Once a week	5
Very rarely or not at all	6

15. How often do you consume fish or seafood?

Very rarely or not at all	1
Once a week	2
Twice a week	3
More than twice a week	4
Daily	5

16. How often do you consume sweets/pastry products?

Daily	1
More than twice a week	2
Twice a week	3
Once a week	4
Very rarely or not at all	5

17. How often do you consume pasta, rice or other grains?

Very rarely or not at all	1
Once a week	2
Twice a week	3
More than twice a week	4
Daily	5

18. How much bread do you consume per day?

More than 12 slices	1
8-12 slices	2
5-7 slices	3
1-4 slices	4
Very rarely or not at all	5

19. How often do you consume dairy products?

Very rarely or not at all	1
Once a week	2
Twice a week	3

More than twice a week	4
Daily	5

20. How many eggs do you consume per week?

Very rarely or not at all	1
1 - 2 eggs	2
3 - 4 eggs	3
5 - 7 eggs	5
More than 7 eggs	4

21. What category of food products do you consume most frequently?

Fast food products	1
Pizza, snacks, pastry, sweets	2
Products made from processed meats and canned goods	3
Food cooked in restaurants	4
Home-cooked meals	5
Special dietary or therapeutic food purchased from specialized units	6

22. What type of cooked food do you consume most frequently?

Fried foods	1
Foods cooked over wood or charcoal	2
Grilled foods	3
Oven-baked foods	4
Boiled or steamed foods	5
Raw or minimally processed foods	6

23. How much water do you drink per day?

Less than 1 liter	1
1 liter	2
2 liters	3
3 liters	4
More than 3 liters	5

24. What category of liquids do you most commonly consume?

Alcoholic beverages: sparkling wine, beer, etc.	1
Carbonated or sweetened non-alcoholic beverages	2
Coffee	3
Tea	4
Still water and natural juices	5

25. Which category of food products predominates in your daily diet? (Multiple options)

Vegetables and fruits	0-if unchecked; 1-if checked
Fish and seafood dishes	0-if unchecked; 1-if checked
Cereals and pasta	0-if unchecked; 1-if checked
Dairy products	0-if unchecked; 1-if checked
Meat and meat products	0-if unchecked; 1-if checked
Sweets and pastry products	0-if unchecked; 1-if checked
High-fat foods	0-if unchecked; 1-if checked
Special foods for cancer patients	0-if unchecked; 1-if checked
Min 1 and Max 8 per respondent	

26. What type of meat do you consume most frequently?

Fish and/or seafood	5
Poultry	4
Rabbit meat	4
Beef	7
Game meat	1
Pork	3

Lamb meat	2
Goat meat	3
Other	1
I do not consume meat	3

27. The choice of food products in your diet and/or dietary supplements is based on information received from: (Multiple options)

Oncologist	0-if unchecked; 1-if checked
Nutritionist	0-if unchecked; 1-if checked
Family doctor	0-if unchecked; 1-if checked
Pharmacist	0-if unchecked; 1-if checked
Medical journals or articles	0-if unchecked; 1-if checked
Acquaintances, friends	0-if unchecked; 1-if checked
Social media	0-if unchecked; 1-if checked
Personal taste	0-if unchecked; 1-if checked
Other	0-if unchecked; 1-if checked
Min 1 and Max 9 per respondent	

Lifestyle:

28. How are your meals distributed throughout the day?

I consume 1-2 meals per day without a fixed schedule	1
I consume 3 meals per day without a fixed schedule	2
I consume 3 meals per day and 1-2 snacks without a fixed schedule	3
I consume 3 meals per day on a fixed schedule	4
I consume 3 meals per day and 1-2 snacks on a fixed schedule	5

29. How do you assess the quality of food you consume daily?

I believe I eat haphazardly, excessively	1
I believe I eat haphazardly, insufficiently	2
I consume food moderately, without excess	3
I consume food based on my body's needs through weight monitoring	4
I consume food based on a ration set by a specialist	5

30. Do you believe your diet has affected your health?

I don't know	1
Yes, because I consume unhealthy foods	2
Yes, because I overeat	3
Yes, because I undereat	4
No	5

31. How do you approach mealtime?

I generally eat in a hurry	1
During meals, I tend to do other things as well	2
I eat my meal calmly and without rushing	3

32. Do you think you need the advice from a nutritionist? (Multiple options)

Yes, to help me choose healthy foods	0-if unchecked; 1-if checked
Yes, to help me eat moderately	0-if unchecked; 1-if checked
Yes, because I need to lose weight urgently	0-if unchecked; 1-if checked
Yes, because I need to gain weight urgently	0-if unchecked; 1-if checked
No	0-if unchecked; 1-if checked
Min 1 and Max 4 per respondent	

33. What type of diet are you currently following?

Normal omnivorous diet	4
Vegetarian diet/variants	2
Vegan diet/variants	3

Ketogenic diet	1
Mediterranean diet	5
Other	0

34. In the last 3 months, have you experienced any changes in your body weight?

Yes, I lost up to 2 kg	1
Yes, I lost 2-4 kg	2
Yes, I lost 4-6 kg	3
Yes, I lost over 6 kg	5
Yes, I gained up to 2 kg	2
Yes, I gained 2-4 kg	1
Yes, I gained 4-6 kg	2
Yes, I gained over 6 kg	3
My weight has remained constant	1

35. Do you currently smoke?

Yes, excessively daily	1
Yes, 1-2 cigarettes daily	2
Yes, 2-3 times a week	3
Yes, occasionally	4
No	5

36. How many hours per night do you usually sleep?

I frequent have insomnia	1
Less than 7 hours per night	2
More than 9 hours a night	3
7-9 hours per night	4

37. Have you been engaging in physical exercise recently?

Yes, 30 minutes daily	4
Yes, at least one hour daily	5
Yes, 2-3 times a week	3
Yes, once a week	2
Yes, very rarely	1
No, due to health conditions	1
No, because I am immobilized	1
No, because I do not usually exercise	1

38. What do you consider to be the main factors affecting your mental state? (Multiple options)

Health problems	0-if unchecked; 1-if checked
Fatigue	0-if unchecked; 1-if checked
Stress	0-if unchecked; 1-if checked
Work-related issues	0-if unchecked; 1-if checked
Lack of communication, isolation	0-if unchecked; 1-if checked
Inadequate social environment	0-if unchecked; 1-if checked
Family problems	0-if unchecked; 1-if checked
Financial problems	0-if unchecked; 1-if checked
None	0-if unchecked; 1-if checked
Min 1 and Max 8 per respondent	

39. What kind of issues do you face? (Multiple options)

I am frequently tired	0-if unchecked; 1-if checked
I am frequently nervous	0-if unchecked; 1-if checked
I am frequently depressed	0-if unchecked; 1-if checked
I am frequently exhausted	0-if unchecked; 1-if checked
I frequently experience panic attacks/anxiety	0-if unchecked; 1-if checked
Emotional eating	0-if unchecked; 1-if checked
Lack of appetite	0-if unchecked; 1-if checked
I am fine, I have no problems	0-if unchecked; 1-if checked

I am frequently tired	0-if unchecked; 1-if checked
Min 1 and Max 8 per respondent	

40. What type of clinical manifestations do you most frequently experience and are most bothersome to you lately? (Multiple options)

Headaches	0-if unchecked; 1-if checked
Dizziness	0-if unchecked; 1-if checked
Physical weakness	0-if unchecked; 1-if checked
Nausea	0-if unchecked; 1-if checked
Dry mouth	0-if unchecked; 1-if checked
Altered sense of smell	0-if unchecked; 1-if checked
Altered sense of taste	0-if unchecked; 1-if checked
Diarrhea	0-if unchecked; 1-if checked
Constipation	0-if unchecked; 1-if checked
Speech difficulties, expression problems	0-if unchecked; 1-if checked
Concentration difficulties, memory problems	0-if unchecked; 1-if checked
Pain	0-if unchecked; 1-if checked
Hair loss	0-if unchecked; 1-if checked
Edema	0-if unchecked; 1-if checked
Breathing difficulties	0-if unchecked; 1-if checked
Locomotor difficulties (walking, manual work)	0-if unchecked; 1-if checked
Eating difficulties (chewing, swallowing)	0-if unchecked; 1-if checked
Low immunity	0-if unchecked; 1-if checked
Injuries, skin rashes, itching	0-if unchecked; 1-if checked
Other	0-if unchecked; 1-if checked
I am fine, I have no problems	0-if unchecked; 1-if checked
Min 1 and Max 20 per respondent	

41. Which of the clinical manifestations have been tolerable for you in the recent period among those listed? (Multiple options)

Headaches	0-if unchecked; 1-if checked
Dizziness	0-if unchecked; 1-if checked
Physical weakness	0-if unchecked; 1-if checked
Nausea	0-if unchecked; 1-if checked
Dry mouth	0-if unchecked; 1-if checked
Altered sense of smell	0-if unchecked; 1-if checked
Altered sense of taste	0-if unchecked; 1-if checked
Diarrhea	0-if unchecked; 1-if checked
Constipation	0-if unchecked; 1-if checked
Speech difficulties, expression problems	0-if unchecked; 1-if checked
Concentration difficulties, memory problems	0-if unchecked; 1-if checked
Pain	0-if unchecked; 1-if checked
Hair loss	0-if unchecked; 1-if checked
Edema	0-if unchecked; 1-if checked
Breathing difficulties	0-if unchecked; 1-if checked
Locomotor difficulties (walking, manual work)	0-if unchecked; 1-if checked
Eating difficulties (chewing, swallowing)	0-if unchecked; 1-if checked
Low immunity	0-if unchecked; 1-if checked
Injuries, skin rashes, itching	0-if unchecked; 1-if checked
I am fine, I have no problems	0-if unchecked; 1-if checked
Min 1 and Max 19 per respondent	

42. Which of the clinical manifestations have not bothered you at all in the recent period among those listed? (Multiple options)

Headaches	0-if unchecked; 1-if checked
Dizziness	0-if unchecked; 1-if checked
Physical weakness	0-if unchecked; 1-if checked

Nausea	0-if unchecked; 1-if checked
Dry mouth	0-if unchecked; 1-if checked
Altered sense of smell	0-if unchecked; 1-if checked
Altered sense of taste	0-if unchecked; 1-if checked
Diarrhea	0-if unchecked; 1-if checked
Constipation	0-if unchecked; 1-if checked
Speech difficulties, expression problems	0-if unchecked; 1-if checked
Concentration difficulties, memory problems	0-if unchecked; 1-if checked
Pain	0-if unchecked; 1-if checked
Hair loss	0-if unchecked; 1-if checked
Edema	0-if unchecked; 1-if checked
Breathing difficulties	0-if unchecked; 1-if checked
Locomotor difficulties (walking, manual work)	0-if unchecked; 1-if checked
Eating difficulties (chewing, swallowing)	0-if unchecked; 1-if checked
Low immunity	0-if unchecked; 1-if checked
Injuries, skin rashes, itching	0-if unchecked; 1-if checked
I am fine, I have no problems	0-if unchecked; 1-if checked
Min 1 and Max 19 per respondent	

43. How do you assess the evolution of your health in the recent period?

It has deteriorated	1
It has improved	3
There has been no change	2

44. What type of therapy have you undergone or are currently undergoing? (Multiple options)

Surgery	0-if unchecked; 1-if checked
Chemotherapy	0-if unchecked; 1-if checked
Immunotherapy	0-if unchecked; 1-if checked
Radiation therapy	0-if unchecked; 1-if checked
Hormonal therapy	0-if unchecked; 1-if checked
Palliative care	0-if unchecked; 1-if checked
Targeted personalized therapy	0-if unchecked; 1-if checked
Other	0-if unchecked; 1-if checked

45. What other treatments have you tried besides oncologic treatment? (Multiple options)

Nutritional supplements	0-if unchecked; 1-if checked
Naturopathic treatments	0-if unchecked; 1-if checked
Acupuncture	0-if unchecked; 1-if checked
Yoga	0-if unchecked; 1-if checked
Others	0-if unchecked; 1-if checked
No other treatment	0-if unchecked; 1-if checked
Min 1 and Max 5 per respondent	

46. How much time do you spend on average each day in front of a computer, tablet phone, or television?

More than 8 hours	1
6-7 hours	2
4-5 hours	3
2-3 hours	4
Less than 1 hour	5

47. What other types of chronic conditions do you have? (Multiple options)

Hypertension	0-if unchecked; 1-if checked
Cardiovascular diseases	0-if unchecked; 1-if checked
Diabetes	0-if unchecked; 1-if checked
Obesity	0-if unchecked; 1-if checked
Lung diseases	0-if unchecked; 1-if checked

Kidney conditions	0-if unchecked; 1-if checked
Autoimmune diseases	0-if unchecked; 1-if checked
Gastric conditions	0-if unchecked; 1-if checked
Liver conditions	0-if unchecked; 1-if checked
Respiratory conditions	0-if unchecked; 1-if checked
Rheumatic conditions	0-if unchecked; 1-if checked
Bone conditions	0-if unchecked; 1-if checked
Mental health conditions	0-if unchecked; 1-if checked
Other	0-if unchecked; 1-if checked
No other conditions	0-if unchecked; 1-if checked
I don't know	0-if unchecked; 1-if checked
Min 1 and Max 15 per respondent	

48. Which of the following situations have you encountered in the past? (Multiple options)

I worked in a polluted environment with high levels of pollutants (chemical, radiation, microorganisms)	0-if unchecked; 1-if checked
I worked with asbestos products or in an asbestos factory	0-if unchecked; 1-if checked
I had excessive sun exposure or used procedures for maintaining a tan	0-if unchecked; 1-if checked
I smoked moderately or excessively	0-if unchecked; 1-if checked
I consumed alcohol moderately or excessively	0-if unchecked; 1-if checked
I used drugs moderately or excessively	0-if unchecked; 1-if checked
I consumed a lot of industrially processed foods (processed meats, pastry products, canned goods) or fast food	0-if unchecked; 1-if checked
I consumed a lot of fried or grilled foods excessively	0-if unchecked; 1-if checked
I consumed a lot of smoked foods	0-if unchecked; 1-if checked
I consumed a lot of carbonated beverages	0-if unchecked; 1-if checked
I consumed few vegetables and fruits	0-if unchecked; 1-if checked
I was overweight for a long time	0-if unchecked; 1-if checked
I worked excessively	0-if unchecked; 1-if checked
I faced many stressful situations	0-if unchecked; 1-if checked
I abused medications	0-if unchecked; 1-if checked
None of the above	0-if unchecked; 1-if checked
Min 1 and Max 15 per respondent	

49. Have you had family members diagnosed with cancer?

Yes	3
No	2
I don’t know	1

50. What do you think is currently lacking in your life to improve your lifestyle? (Multiple options)

Free time	0-if unchecked; 1-if checked
Restful sleep, relaxation	0-if unchecked; 1-if checked
Financial resources	0-if unchecked; 1-if checked
Guidance from a nutrition specialist	0-if unchecked; 1-if checked
Psychological counseling	0-if unchecked; 1-if checked
Access to quality medical services	0-if unchecked; 1-if checked
Resolution of stressful problems	0-if unchecked; 1-if checked
Communication and socialization	0-if unchecked; 1-if checked
Reducing time spent on social media or various websites	0-if unchecked; 1-if checked
Knowledge in the field of nutrition	0-if unchecked; 1-if checked
Exercise, physical activity	0-if unchecked; 1-if checked

Quality food products	0-if unchecked; 1-if checked
Someone to help me whenever I need it	0-if unchecked; 1-if checked
Nothing	0-if unchecked; 1-if checked
Min 1 and Max 13 per respondent	

51. How are your oncologist appointments provided?

Free through the public healthcare system	1
For a fee through the private healthcare system	2
Both modalities	3

52. How easily can you reach your doctor from the moment you contact them?

On the same day	4
Within a few days	3
Within a week	2
More than a week	1

53. To what extend do you follow your current doctor's recommendations?

Completely	2
Partially	1

54. How often do you have a medical consultation?

Monthly	5
Every 6 months	4
Every 3 months	3
Annually	2
More than a year	1

55. How do you spend your leisure time? (Multiple options)

Watching movies and TV programs	0-if unchecked; 1-if checked
On social media or various websites	0-if unchecked; 1-if checked
Reading	0-if unchecked; 1-if checked
DIY projects	0-if unchecked; 1-if checked
With family or friends	0-if unchecked; 1-if checked
Outdoor activities	0-if unchecked; 1-if checked
Participating in cultural activities	0-if unchecked; 1-if checked
Participating in charitable events	0-if unchecked; 1-if checked
Participating in training courses	0-if unchecked; 1-if checked
Other ways	0-if unchecked; 1-if checked

56. What type of oncological pathology do you have? (One option)

Lung cancer	1
Liver cancer	2
Gastric cancer	3
Breast cancer	4
Colon cancer	5
Pancreatic cancer	6
Prostate cancer	7
Kidney cancer	8
Thyroid cancer	9
Other	10

The Overall attitudinal score is the mean of the scores on all ten items (total the fifty-six items and divide by 56)

The questionary include 7 subscales, scored as followed

- **Adherence to a healthy diet** – mean of items 9-24, 26. These questions assess dietary patterns and specific food preferences:
- **Nutritional Assessment and Diet Management:** mean of questions 28-30, 32-34. These questions assess meal frequency, dietary satisfaction, perceptions of dietary impact on health, dietary advice needs, and changes in body weight. This subscale

focuses on how patients manage their nutrition in the context of their overall health and specific dietary requirements.

- **Physical Health Management:** mean of questions 35-37, which covers individual habits such as smoking and sleep patterns, along with physical exercise habits (assuming any single-response aspects). This subscale is crucial for understanding the physical aspects of health that are directly manageable through lifestyle choices.
- **Treatment Adherence and Medical Interaction:** mean of questions 43, 53, 54. These questions are focused on the types of therapies patients are undergoing, their adherence to doctor's recommendations, and the frequency of medical consultations. This subscale is important for monitoring how patients are following through with their prescribed treatments and their ongoing interaction with healthcare providers.
- **Support Systems and Access to Care:** mean of questions 49-52: These questions Investigate family health history, perceived gaps in lifestyle needs, details about oncologist appointments, and accessibility of medical care. This section delves into the support structure available to the patient, including family influences and healthcare system interactions, which are vital for comprehensive patient care.
- **Multiple-Answer Selection Type Questions:** For questions allowing multiple responses, scores are calculated based on the percentage of respondents selecting each option: 25, 27, 31, 32, 37, 38, 39, 40, 41, 42, 44, 45, 46, 42, 46, 47, 48, 50, 55 (predominant food categories in diet, approach to mealtime, need for nutritional advice, physical exercise engagement, clinical manifestations experienced and their impact).

$$\frac{Number\ of\ responses\ for\ answer\ choice}{Number\ of\ total\ responses} \times 100$$

- the value of Cronbach's α was 0.815, what it indicates is a good internal consistency and that the scale is reliable.

1. Hamed Taherdoost. Designing a Questionnaire for a Research Paper: A Comprehensive Guide to Design and Develop an Effective Questionnaire. Asian Journal of Managerial Science, 2022, 11, pp.8-16. doi:10.51983/ajms-2022.11.1.3087.hal-0374183
2. James BL, Loken E, Roe LS, Myrissa K, Lawton CL, Dye L, Rolls BJ. Validation of the Diet Satisfaction Questionnaire: a new measure of satisfaction with diets for weight management. Obes Sci Pract. 2018 Oct 10;4(6):506-514. doi: 10.1002/osp4.299. PMID: 30574344; PMCID: PMC6298208.
3. Dubasi SK, Ranjan P, Arora C, Vikram NK, Dwivedi SN, Singh N, Kaloiya GS, Shalimar. Questionnaire to assess adherence to diet and exercise advices for weight management in lifestyle-related diseases. J Family Med Prim Care. 2019 Feb;8(2):689-694. doi: 10.4103/jfmpc.jfmpc_338_18. PMID: 30984696; PMCID: PMC6436250.
